# Respiratory Syncytial Virus (RSV) Neutralizing Antibodies at Birth Predict Protection from RSV Illness in Infants in the First 3 Months of Life

**DOI:** 10.1093/cid/ciaa648

**Published:** 2020-05-28

**Authors:** Andrea G Buchwald, Barney S Graham, Awa Traore, Fadima Cheick Haidara, Man Chen, Kaitlyn Morabito, Bob C Lin, Samba O Sow, Myron M Levine, Marcela F Pasetti, Milagritos D Tapia

**Affiliations:** 1 Department of Environmental and Occupational Health, Colorado School of Public Health, Denver, Colorado, USA; 2 Vaccine Research Center, National Institute of Allergy and Infectious Diseases, National Institutes of Health, Bethesda, Maryland, USA; 3 Centre pour le Développement des Vaccins–Mali, Bamako, Mali; 4 Department of Pediatrics, Center for Vaccine Development and Global Health, University of Maryland School of Medicine, Baltimore, Maryland, USA

**Keywords:** respiratory syncytial virus, maternal antibodies, protective threshold

## Abstract

**Background:**

Respiratory syncytial virus (RSV) is a leading cause of viral pneumonia and bronchiolitis during the first 6 months of life. Placentally transferred antibodies can prevent severe RSV illness, and maternal immunization may reduce illness in young infants. Identification of protective antibody levels facilitates the advancement of vaccine candidates and maternal immunization.

**Methods:**

We conducted a nested case-control study with 587 Malian mother–infant pairs, followed from birth to age 6 months. RSV cases were infants who developed influenza-like illness (ILI) or pneumonia and were RSV-positive by polymerase chain reaction. Cases were matched to healthy controls and RSV-negative ILI controls. RSV-A and RSV-B neutralizing antibodies were measured in maternal, cord blood, and infant sera at age 3 and 6 months.

**Results:**

Maternal antibodies were efficiently transferred to infants. Maternal and infant RSV titers were strongly correlated. Infant antibody titers against RSV-A were 3 times higher than those against RSV-B. At birth, infants who remained healthy had significantly higher RSV-A and RSV-B titers compared with infants who subsequently contracted RSV. RSV-A inhibitory concentration (IC)_80_ titer >239 or RSV-B titer >60 at birth was significantly associated with being a healthy control compared with an RSV case within the first 3 months of life. RSV-A IC_80_ titers in cord blood were associated with decreased episodes of pneumonia.

**Conclusions:**

Maternally acquired RSV antibodies were associated with protection of infants against community-detected cases of RSV-ILI and pneumonia. RSV titers in cord blood can predict whether an infant will be infected with RSV or remain uninfected.

## Teaser

Maternally acquired respiratory syncytial virus (RSV) neutralizing antibodies were associated with infant protection against RSV up to 3 months. These findings indicate that RSV titers at birth can predict whether an infant will be infected with RSV or remain uninfected in early life.

Respiratory syncytial virus (RSV) is a leading cause of viral pneumonia and bronchiolitis among children aged <5 years worldwide [1]. Pneumonia before age 6 months is a frequent cause of early childhood mortality [2]. While RSV-related infant death remains a major problem in developed nations, 99% of RSV-related deaths occur in low-resource settings [3]. There are limited treatment options for RSV, and no vaccine is available to protect infants from RSV infection. However, it has been shown that RSV transplacental antibody transfer from mother to infant is efficient, suggesting that maternal vaccination in the early third trimester may be effective for protecting infants early in life [4–6].

Previous studies have shown that RSV neutralizing antibody (nAb) levels in cord blood are associated with protection from RSV hospitalization among children aged <6 months [7, 8]. Additionally, the severity of RSV disease and pneumonia has been associated with RSV-specific antibody levels [9, 10]. There is evidence that maternally derived RSV-specific antibodies can protect against RSV illness and RSV-related lower respiratory infections (LRI). However, analyses from 2 longitudinal cohort studies showed no association between cord blood serum RSV-specific antibody level and RSV LRI [11, 12].

Maternal immunization against RSV may be an effective measure to simultaneously protect mothers against RSV and decrease the risk of illness in infants. Defining protective antibody levels in newborns is necessary to predict the potential efficacy of any maternal immunization strategy. Using a nested case-control study design, we aimed to assess the association between subtype-specific RSV neutralization activity and protection from RSV in the first 6 months of life and identify protective thresholds of maternally derived subtype-specific anti-RSV antibodies at birth.

## METHODS

### Clinical Specimens

To assess the association between RSV neutralization and RSV illness, we used serum and respiratory illness surveillance data from a study on the efficacy of maternal influenza vaccination on influenza risk in infants [13]. The study was conducted in Mali and included pregnant women in their third trimester who received either trivalent influenza vaccine or quadrivalent meningococcal conjugate vaccine.

From September 2011 to April 2013, 4193 enrolled pregnant women were followed with weekly home visits, and their infant(s) was followed up to age 6 months for influenza-like illness (ILI) as outlined previously [13]. The infants were additionally followed for pneumonia, including severe and very severe (as defined by World Health Organization criteria) [14]. Case definitions are included in the Supplementary Materials.

In this study, we used a subset of influenza-negative specimens taken at the time of ILI or pneumonia from infants whose ILI or pneumonia occurred during the months of October 2012–October 2013 based on known RSV circulation in the study area (A. Driscoll, personal oral communication, 2017). The specimens included all samples from infants who presented with fever without an apparent source, 30% of samples collected from infants who presented with fever and acute upper respiratory infection, and all samples collected from infants with pneumonia. Preference was given to cases where the infant was hospitalized or the outcome resulted in death. Specimens included maternal serum at delivery, cord blood, and infant sera at age 3 and 6 months.

### RSV Diagnostic

RSV was detected with the respiratory pathogens 33 real time-PCR polymerase chain reaction kit (Fast Track Diagnostics; Junglinster, Luxembourg). Positive samples were further tested with the RealStar RSV RT-PCR kit (Altona Diagnostics) for RSV-A and RSV-B differentiation.

### Study Design

A nested case-control study design was used and described in detail previously [15]. Briefly, participants were selected from those included in RSV testing described above. Cases were infants who experienced pneumonia or ILI and samples from the episode tested positive for RSV by PCR. Cases were matched by birth date to 2 sets of controls: infants with RSV-negative ILI (ILI controls) and infants without any ILI during the study (healthy controls). ILI controls were infants who experienced pneumonia or ILI within the study, received an RSV test for all episodes of pneumonia or ILI, and never tested positive for RSV. Up to 2 ILI controls were matched to each case. Healthy controls were matched to cases as a simple random sample of remaining eligible controls after ILI controls had been assigned. A given control could not be matched to more than 1 case. The matches were made in chronological order, according to the date of RSV case fever onset and RSV case age.

### RSV Neutralization Assays

Fluorescently (mKate)-labeled recombinant RSV-expressing F genes from subtype A or B were incubated with serially diluted serum samples, and the mix was added to Hep 2 cells. Following incubation, cells were lysed and fluorescence intensity determined. Detailed methods are included in the Supplementary Materials. The 50% and 80% inhibitory concentrations (IC_50_ and IC_80_) for each sample were calculated using 5-parameter curve fitting and nonlinear regression. The limit of detection of the assay was 20 (inverse of starting dilution); sera that did not reach 50% or 80% inhibition at the 1:20 dilution were assigned a titer of 10.

### Statistical Analyses

Median and interquartile range were calculated for RSV nAb levels (IC_80_ and IC_50_ for both RSV-A and RSV-B) at birth for samples from mothers and infants by group. The mother-to-infant ratio was also calculated for each group. Cases were analyzed both by RSV subtype and as a single pooled group. The geometric mean neutralizing activity was calculated for infants at zero, 3, and 6 months. Preliminary analysis compared antibody distribution between groups using nonparametric Wilcoxon rank sum tests.

Matched analysis to account for confounding by case age and date was done using conditional logistic regression, comparing controls to RSV cases. Three distinct matched analyses were done for both RSV-A neutralization (IC_80_) and RSV-B neutralization (IC_80_): using IC_80_ in cord blood as the predictor of interest, using maternal neutralizing activity at birth as the predictor, and stratified by timing of RSV case, with cord blood neutralizing activity compared among matched infants where RSV cases occurred before age 3 months and neutralizing activity at 3 months compared among infants where RSV cases occurred after age 3 months.

To define a cord blood neutralizing activity IC_80_ level that can predict an infant being a healthy control, the sensitivity and specificity of IC_80_ levels for determining healthy control status were calculated. The IC_80_ value with the maximum sensitivity + specificity level, or the minimum value of *d* (distance from the upper left corner of the receiver operating characteristic curve), was used to define cutoff points for predictive values of RSV-A IC_80_ and RSV-B IC_80_.

We hypothesized that the association between ILI controls and cases could be confounded by overall poor health among ILI controls. To account for this, maternal socioeconomic status, household crowding, infant birth weight, and gestational age were examined as potential confounders by first assessing the bivariate association with case status and later testing for inclusion in conditional logistic regression models.

The same analyses were performed for RSV IC_50_. All analyses are summarized in Supplementary Table S0 and were conducted using SAS 9.4.

## RESULTS

### Maternal–Infant Pairs and RSV-Antibody Transfer

Among infants included [15], 157 had ILI episodes that tested positive for RSV; among these, 146 infants had cord blood samples for serological analysis (RSV cases). Of these 146 infants, 27 had ILI episodes that tested positive for RSV-A and 124 for RSV-B. Five ILI episodes tested positive for both RSV-A and RSV-B. RSV cases were matched to 190 infants who had ILI and tested negative for RSV (ILI controls) and 251 infants who did not experience any ILI (healthy controls). There were no significant differences between mothers at baseline (Table 1).

**Table 1.** Demographic and Clinical Characteristics of Mothers at Baseline, by Case Status

Characteristic	RSV Cases (N = 146)	ILI Controls (N = 190)	Healthy Controls (N = 251)
Maternal characteristics			
Socioeconomic index,^a^ mean (SD)	0.65 (3.5)	−0.09 (3.5)	0.46 (3.8)
Less than primary education, n (%)	120 (82)	156 (82)	199 (79)
Primary or more education, n (%)	26 (18)	34 (18)	52 (21)
Household crowding, people/room, mean (SD)	2.8 (2.1)	2.8 (1.7)	2.8 (1.5)
Age, mean (SD), y	25.6 (6.2)	24.9 (5.9)	24.0 (6.0)
Infant characteristics			
Gestational age, mean (SD), weeks	39.9 (2.2)	39.4 (2.3)	39.7 (2.3)
<37 weeks (preterm), n (%)	11 (7)	11 (6)	15 (6)
Birth weight, mean (SD), g	3124 (476)	3063 (453)	3068 (432)
<2500 g (low birth weight), n (%)	8 (5)	21 (11)	25 (4)
ILI cases, n	117	173	–
Pneumonia cases, n	29	17	–
RSV-A cases, n	27	–	–
First 3 months, ILI/pneumonia	8/2	–	–
RSV-B cases, n	124	–	–
First 3 months, ILI/pneumonia	27/12	–	–

Abbreviations: ILI, influenza-like illness; RSV, respiratory syncytial virus; SD, standard deviation; –, not applicable.

^**a**^Socioeconomic index has population mean = 0.33, SD = 3.67, range = (−7 to 13).

**Figure 1.**
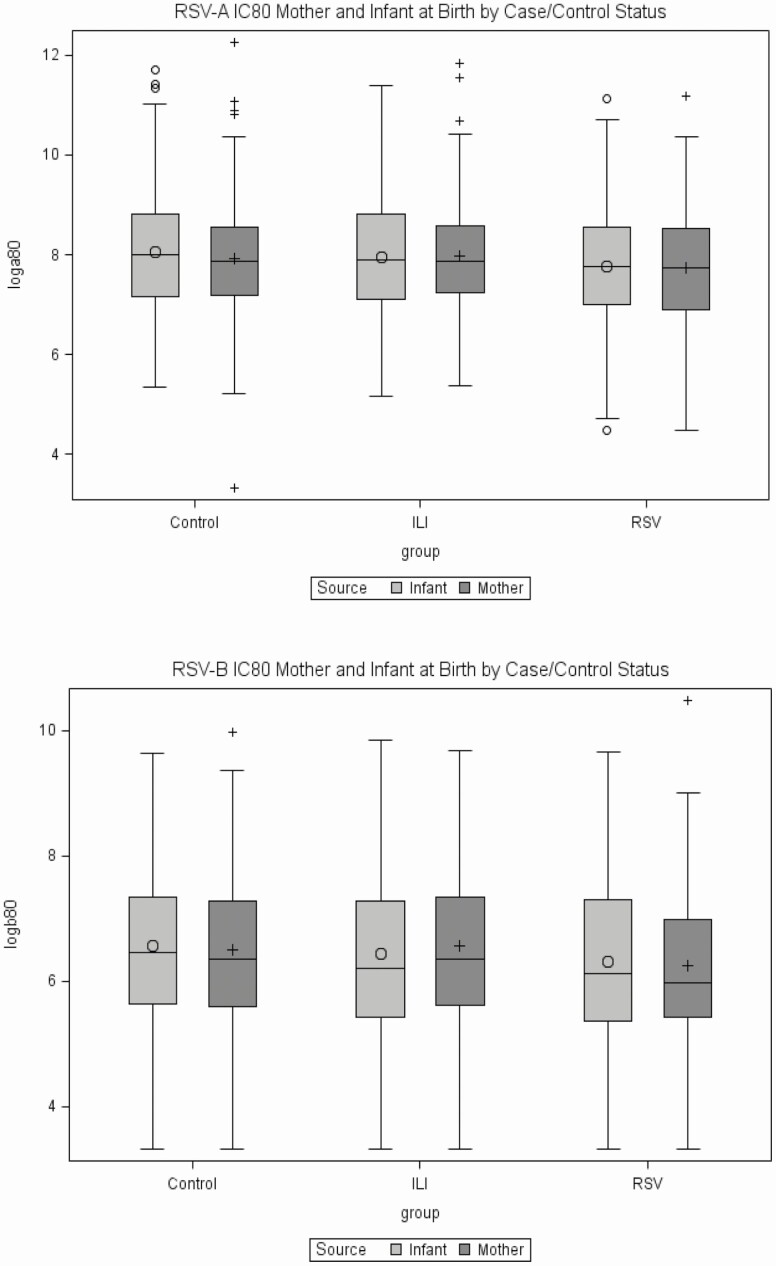
Maternal and infant RSV neutralizing activity (IC_80_) at birth. (*A*) RSV-A and (*B*) RSV-B. Data show log transformed (base 2) reciprocal IC_80_ sera dilution for mothers and infants in healthy controls, ILI (RSV neg), and RSV (ILI pos) groups. Abbreviations: IC_80_, 80% inhibitory concentration; ILI, influenza-like illness; RSV, respiratory syncytial virus.

Maternal and infant subtype-specific nAb at birth is shown in Figure 1. The median infant-to-maternal nAb activity (IC_80_) ratio at birth was between 1.01 and 1.15 for all groups, except for RSV-B among ILI controls, for which it was 0.93 (Supplementary Table S1). Maternal and infant nAb levels were strongly associated. Correlation between mother–infant IC_80_ titers was the strongest for RSV-B and in ILI controls (Supplementary Table S1).

### Association Between Infant Antibodies at Birth and RSV Disease

Initial unmatched analysis of neutralizing activity in cord blood for all and subtype-specific cases (Table 2) showed no difference between RSV cases and ILI controls, although a significant difference was found between RSV cases and healthy controls. Healthy control infants had significantly higher RSV-A and RSV-B neutralizing activity at birth compared with RSV cases regardless of the subtype (Table 2). In all groups, infant neutralizing titers against RSV-A were 3 times higher than those against RSV-B (Table 2 and Supplementary Table S2).

**Table 2.** Cord Blood Samples, Median and Interquartile Range of Respiratory Syncytial Virus (RSV)-A and RSV-B Neutralization (80% Inhibitory Concentration) by Group; *P* Values Comparing RSV Cases (All and Strain Specific) vs Controls

Group	All Cases (N = 146)		Strain-specific Cases	
	**Cord Blood IC**_**80**_ **Median (IQR)**	***P* Value (Wilcoxon)**	**Cord Blood IC**_**80**_ **Median (IQR)**	***P* Value (Wilcoxon)**
RSV-A IC_80_ levels				
RSV cases	217 (128–377)	Ref	219 (108–530) (n = 27)	Ref
ILI controls	240 (137–453)	.22	240 (137–453)	.62
Healthy controls	256 (143–447)	.04	256 (143–447)	.32
RSV-B IC_80_ levels				
RSV cases	70 (41–159)	Ref	72 (44–127) (n = 124)	Ref
ILI controls	75 (44–156)	.47	75 (44–156)	.54
Healthy controls	88 (51–163)	.03	88 (51–163)	.04

Abbreviations: IC_80_, 80% inhibitory concentration; ILI, influenza-like illness; IQR, interquartile range; Ref, reference group; RSV, respiratory syncytial virus.

^a^ILI controls and healthy controls were compared with RSV cases in unmatched preliminary analysis using nonparametric Wilcoxon rank sum tests.

**Figure 2.**
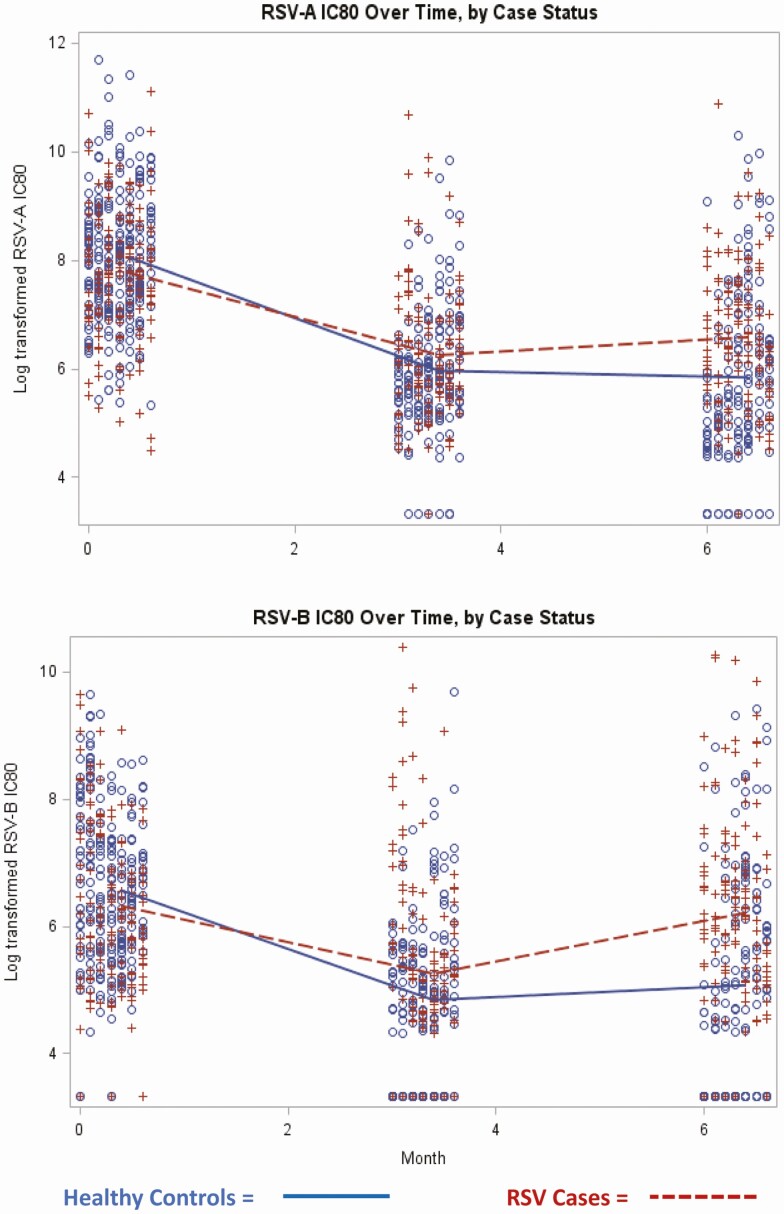
Infant RSV neutralizing antibody (IC_80_) kinetics by case status. (*A*) RSV-A and (*B*) RSV-B. Data show log transformed (base 2) titers in RSV cases and healthy controls. (+) indicate RSV cases and (o) indicate healthy controls. Abbreviations: IC_80_, 80% inhibitory concentration; RSV, respiratory syncytial virus.

Among potential confounders examined for the association between case status and neutralizing activity at birth, only gestational age was associated with RSV-B nAb activity. None of the variables examined were associated with case status, and thus no variables were included as confounders for additional analyses. Neutralizing activity decreased significantly (to detection limit) at 3 months in all groups (Figure 2, Supplementary Table S2). Levels remained similar at age 6 months.

### Timing of RSV Illness

Next, we investigated the association between nAb levels and case status by the timing of RSV infection. In preliminary analyses, cases with any RSV infection (Table 3) or with RSV-A infection (Supplementary Table S4) in the first 3 months had significantly lower RSV-A IC_80_ levels at birth compared with healthy controls. There was also a trend of lower IC_80_ levels among infants with early RSV infection compared with ILI controls. No difference in antibody titer was detected for RSV-B between cases and controls and between later cases of RSV and controls (Table 3).

**Table 3.** Neutralizing Activity (80% Inhibitory Concentration) Before Influenza-like Illness (ILI) Stratified by Timing of ILI: Comparing Controls With All Respiratory Syncytial Virus Cases

Group	Cases Up to 90 Days		Cases After 90 Days	
	**Cord Blood IC**_**80**_ **, Median (IQR)**	***P* Value** **(Wilcoxon)**	**3 Month IC**_**80**_ **, Median (IQR)**	***P* Value (Wilcoxon)**
RSV-A IC_80_ levels				
RSV cases	170.1 (127.6–335.0)	Ref (n = 47)	55.0 (44.8–87.3)	Ref (n = 93)
ILI controls	241.2 (134.0–485.4)	.10	52.5 (37.9–91.4)	.49
Healthy controls	259.0 (143.0–447.0)	.03	58.9 (40.3–99.4)	.63
RSV-B IC_80_ levels				
RSV cases	77.6 (42.1–214.8)	Ref (n = 46)	34.5 (26.5–48.7)	Ref (n = 91)
ILI controls	75.6 (43.4–155.9)	.70	34.8 (27.1–46.2)	.80
Healthy controls	88.9 (51.3–163.2)	.62	39.2 (26.9–57.5)	.35

Abbreviations: IC_80_, 80% inhibitory concentration; ILI, influenza-like illness; IQR, interquartile range; Ref, reference group; RSV, respiratory syncytial virus.

^a^ILI controls and healthy controls were compared with RSV cases in unmatched preliminary analysis using nonparametric Wilcoxon rank sum tests.

### Adjusted Analysis—Controlling for Matching

Final adjusted analysis, controlling for matching, was done using conditional logistic regression with log-transformed IC_80_ titers as the predictor variable and case status as the outcome. Before age 3 months, higher RSV-A IC_80_ neutralizing activity was associated with nonsignificant protection from RSV-A (Supplementary Table S6), and higher RSV-A antibodies were significantly predictive of being a healthy control compared with having any RSV infection, with a 10-fold increase in the RSV-A IC_80_ being associated with an 84% increase in the odds of being a healthy control (Table 4).

**Table 4.** Infant Samples, Matched Analysis Using Conditional Logistic Regression for Odds of Being a Control Compared With All Respiratory Syncytial Virus Cases (With Any Strain) Stratified by Timing of Case

Group	Case Before 3 Months		Case After 3 Months	
	**OR** ^**a**^	***P* Value**	**OR** ^**a**^	***P* Value**
Comparing RSV-A IC_80_				
RSV cases	1.00	Ref (n = 47)	1.00	Ref (n = 93)
ILI controls	1.45 (0.87–2.43)	.15	0.65 (0.38–1.10)	.11
Healthy controls	1.84 (1.09–3.10)	0.02	1.00 (0.67–1.50)	.99
Comparing RSV-B IC_80_				
RSV cases	1.00	Ref (n = 46)	1.00	Ref (n = 91)
ILI controls	0.81 (0.50–1.31)	.40	0.46 0.19 (1.11)	.09
Healthy controls	1.41 (0.87–2.30)	.16	0.83 (0.40–1.73)	.62

Abbreviations: IC_80_, 80% inhibitory concentration; ILI, influenza-like illness; OR, odds ratio; Ref, reference group; RSV, respiratory syncytial virus.

^**a**^OR for a 1 natural log increase in IC_80_ among infant samples.

Using the IC_80_ value with the maximum sensitivity + specificity level for predicting RSV illness, cut-points identified were RSV-A IC_80_ = 239 or greater and RSV-B IC_80_ = 60 or greater. Looking at IC_80_ titers as a continuous variable, there was no difference in RSV-A antibody levels at birth between ILI controls and RSV cases before age 3 months (Table 4). However, after dichotomizing nAb levels as low/high using the chosen predictive value of RSV-A IC_80_ = 239, having RSV-A nAb titers above 239 at birth was associated with 3 times the odds of being an ILI control compared with an RSV case (Table 5). Looking at RSV-B IC_80_ neutralization as a continuous variable, there was no association between RSV-B antibody levels and case status (Table 4). However, after dichotomizing RSV-B IC_80_ neutralization, having high RSV-B IC^80^ greater than 60 in cord blood was significantly associated with being a healthy control compared with all RSV cases (Table 5).

**Table 5.** Infant Samples, Matched Analysis Using Conditional Logistic Regression, Stratified by Timing of Case, Neutralizing Activity (80% Inhibitory Concentration) Dichotomized High (Respiratory Syncytial Virus [RSV]-A ≥239, RSV-B ≥60)/low

Group	Case Before 3 Months		Case After 3 Months	
	**OR** ^**a**^ **(95% CI)**	***P* Value**	**OR** ^**a**^ **(95% CI)**	***P* Value**
Comparing RSV-A IC_80_ high/low				
RSV cases	1.00	Ref (n = 47)	1.00	Ref (n = 93)
ILI controls	3.15 (1.28–7.79)	.01	0.76 (.22–2.67)	.67
Healthy controls	4.22 (1.67–10.67)	.002	1.69 (.57–5.01)	.35
Comparing RSV-B IC_80_ high/low				
RSV cases	1.00	Ref (n = 46)	1.00	Ref (n = 91)
ILI controls	0.86 (.41–2.78)	.72	0.74 (.49–1.78)	.39
Healthy controls	3.94 (1.22–12.62)	.02	0.80 (.25–2.50)	.70

Abbreviations: IC_80_, 80% inhibitory concentration; ILI, influenza-like illness; OR, odds ratio; Ref, reference group; RSV, respiratory syncytial virus.

^**a**^OR for having high IC_80_ among controls compared with all RSV cases

### Association Between Maternal Antibodies and RSV Disease

In initial unmatched analysis, maternal nAb levels at birth were associated with an infant being an RSV-B case compared with ILI or healthy controls (Supplementary Table S8). This association did not remain when accounting for matching in conditional logistic regression (Supplementary Table S9). Further, there was no association between maternal neutralization and being an RSV case when looking only at cases that occurred before age 3 months (Supplementary Table S10).

### Association Between Infant Antibodies and Pneumonia

Last, we determined whether neutralization was predictive of severe disease. There were 46 infants in the dataset who had been hospitalized for pneumonia. In conditional logistic regression, increasing RSV-A IC_80_ neutralizing activity in cord blood was associated with significantly decreased odds of pneumonia (odds ratio [OR], 0.63; 95% confidence interval [CI], .41–.96), and increasing RSV-B IC_80_ neutralizing activity in cord blood was associated with a borderline decrease in odds of pneumonia (OR, 0.67; 95% CI, .41–1.10).

Similar analyses were conducted using neutralizing activity reported as IC_50_ (Supplementary Tables S3, S5, S7). Although results showed similar trends, calculation using IC_50_ titers did not reach significant associations except for the infant antibody levels in cord blood and pneumonia.

### RSV Neutralizing Activity Half-life

The half-life of nAb was calculated for controls through age 12 weeks. Among children without RSV infections, the mean decrease in log2 RSV-A titers was 0.047 (standard deviation [SD] = 0.007)/day, indicating a half-life of 21.3 days. The mean decrease in log2 RSV-B titers was 0.037 (SD = 0.007)/day, giving a half-life of 27.0 days.

## DISCUSSION

We are the first to demonstrate that maternally acquired RSV-specific neutralizing antibodies can protect infants against community-detected cases of RSV-ILI. We are also the first to demonstrate the association between subtype-specific nAb and protection from RSV illness in infants. We aimed to assess the association between RSV nAb levels and protection from RSV in the first 6 months of life and to identify protective thresholds of maternally derived anti-RSV antibodies in cord blood. Using a nested case-control study, we found a significant association between RSV-A nAb levels at birth and protection from RSV-ILI in the first 3 months of life. RSV-A nAb levels were also associated with protection against pneumonia in the first 6 months of life. These findings provide critical evidence supporting maternal vaccination against RSV to protect infants during early infancy.

Several studies have addressed the protective role of maternal antibodies against RSV. However, it is difficult to compare results between studies due to a lack of standardized results. One previous study, which found higher maternal immunoglobulin G (IgG) among mothers whose infants did not have RSV infections, did not evaluate functional activity [16]. Most previous research that examined maternally derived RSV neutralizing antibodies and the risk of disease focused on severe RSV, detected through hospital surveillance or at health clinics [9, 17–19]. While RSV hospitalizations should be a more sensitive outcome than the outcome we used, previous evidence for a protective effect of RSV antibodies at birth have been mixed. In Texas, researchers found that cord blood titers for RSV nAb were significantly higher in the general population than among infants hospitalized for RSV-associated illness [9]. However, studies in Turkey, Kenya, and rural Alaska that looked at hospital-detected RSV were unable to find significant protection associated with cord blood antibody neutralization [17–19]. This may be related to the nAb assays used, the lack of subtype-specific analysis, or low power, as few outcomes were detected. Additionally, use of hospital-detected cases may lead to bias toward the null due to outcome misclassification, as many infants in the population likely experienced RSV infections but were not identified as cases. We may have missed some RSV cases, particularly those without fever or asymptomatic infections [20]. However, this would have biased our results toward the null, supporting the observed significant differences between cases and controls.

In Nepal, the only previous study with actively detected RSV cases, no association was found between cord blood nAb titers and RSV-ILI [12]. This may have been due to insufficient sample size, with only 30 RSV-ILI cases identified.

We are the first to look at subtype-specific RSV antibodies in the context of infection. We found predominantly RSV-B infections, and RSV-B neutralizing activity was lower than that against RSV-A. This could indicate that the RSV-B strain used was less sensitive to neutralization than the A strain or this may be a consequence of prior immunity to RSV-A in the population. We showed, for the first time, an association between RSV-A neutralizing activity and protection against all RSV infections. The association with all cases, as opposed to subtype specificity, suggests potential antibody cross-reactivity [21]. The robust association between maternal and infant antibodies suggests a protective role for maternal IgG against infant RSV. Maternal anti-RSV IgG and infant anti-RSV IgG were not correlated in a previous report [17] that did not analyze functional activity. Virus neutralization is a more specific readout and reveals antibody features and interactions missed by IgG enzyme-linked immunosorbent assays. Antibody neutralization measured by IC_80_ was more sensitive than when measured by IC_50_ and revealed associations and a predictive threshold of protection, which further illustrates the relevance of the methods applied.

We were unable to identify a significant difference between ILI controls and RSV cases in most analyses. ILI controls had the lowest infant-to-maternal ratio of RSV-B neutralizing activity in the population, suggestive of predisposition to poor health or other unmeasured confounders. However, our use of a robust study design, nested case control within a clinical trial, allowed us to identify sufficient numbers of cases and improves the strength of our inferences. Large numbers of cases allowed us to look at infections that occurred in the first 3 months of life, as previously recommended [22]. Due to frequent active follow-up, we are confident that we identified most RSV cases in our population, limiting bias due to misclassification. The half-life of RSV antibodies in our study ranged from 21 to 27 days. These values are consistent with the RSV neutralization half-life of 27 days previously reported at 10 weeks of age in Bangladeshi infants [4], supporting the relevance of our findings.

Our results provide strong evidence of the potential for a vaccine against RSV delivered to pregnant women to protect infants against RSV infection in early infancy. The data also suggest that protection from RSV disease is related to antibody properties, including neutralizing activity and subtype specificity, and may be more apparent when the disease end point is more severe. Maternal vaccination is expected to induce significantly higher maternal anti-RSV IgG levels at birth than those seen in this study. As maternal antibodies have a predictable decay time, vaccine-derived antibodies are expected to protect infants for at least 6 months, when infant vaccination would be appropriate. These data also support birth-dose administration to newborns of novel monoclonal antibodies engineered to achieve protective serum neutralizing activity for up to 5–6 months, a new technology being proposed as an alternative to immunization [23]. Ongoing studies involve analysis of epitope-specific antibody binding patterns, Fc-mediated functional activity, and antibody glycosylation profiles. Epitope-specific neutralizing activity will also be assessed based on the underlying premise that defining antibody specificity and function with more precision will guide future strategies for maternal immunization.

## Supplementary Data

Supplementary materials are available at *Clinical Infectious Diseases* online. Consisting of data provided by the authors to benefit the reader, the posted materials are not copyedited and are the sole responsibility of the authors, so questions or comments should be addressed to the corresponding author.

ciaa648_suppl_Supplementary_Material

## Notes

***Acknowledgments.*** The authors thank Christopher Moore, Lori Romaine, and Consolee Karangwa for assay setup and Bob Bailer for assay supervision.

***Financial support.*** This study was supported by the Bill & Melinda Gates Foundation (grant OPP1002744). This study was supported, in part, by intramural funding from the National Institutes of Allergy and Infectious Diseases, National Institutes of Health.

***Potential conflicts of interest.*** The authors: No reported conflicts of interest. All authors have submitted the ICMJE Form for Disclosure of Potential Conflicts of Interest.
