# Supplementary material for: Respiratory Syncytial Virus (RSV) Neutralizing Antibodies at Birth Predict Protection from RSV Illness in Infants in the First 3 Months of Life
Source: Clin Infect Dis. 2020 May 28;73(11):e4421–7. doi: 10.1093/cid/ciaa648 (PMC8662775; doi:10.1093/cid/ciaa648)
Supplement: ciaa648_suppl_Supplementary_Material [file ciaa648_suppl_supplementary_material.docx]

**METHODS**

**CASE DEFINITIONS**

Influenza-like illness:

Either of the 2 following conditions reported by the caretaker or observed by a clinician:

- Fever without an apparent source, documented by a clinician’s measurement to be an axillary temperature >38°C or maternal perception of fever and administration of antipyretic in previous 8 hours

* No source means that there is no apparent cause for the fever such as soft tissue infection, although generalized symptoms such as irritability, loss of appetite, and/or lethargy may be present;

OR

- Fever (as defined below) plus acute respiratory infection.

Acute respiratory infection is defined as ANY of the following on the same or consecutive days: runny nose, nasal congestion, cough, difficulty breathing, pus draining from ear or wheezing;

PLUS

- 7 days after last reported fever

Fever was defined as any of the following:

- Mother’s perception that the infant had fever during the previous 24 hours
- Mother measured the infant’s temperature as >38°C during the previous 24 hours
- Clinician or study staff measure the infant’s temperature to be >38°C
- Maternal perception of fever and administration of antipyretic in previous 8 hours

Pneumonia:

Infants were defined as having pneumonia if they fit the WHO definition for pneumonia, including severe and very severe pneumonia: Cough or difficulty breathing plus any danger sign (unable to drink/nurse, vomits everything, convulsions, lethargy or unconsciousness) or lower chest indrawing or stridor.

**RSV NEUTRALIZATION ASSAYS**

Recombinant mKate-RSV expressing prototypic F genes from subtype A (strain A2) or subtype B (strain 18537) were titrated to produce a 100-fold fluorescence intensity range between the cells only control and virus and cell only control.

Sera was diluted 5-fold from 1:20 to 1:156,2500 and mixed with an equal volume of titrated recombinant mKate-RSV A or mKate-RSV B and incubated at 37ºC for 45min-1h. Next, 50μl of each mixture was added to HEp-2 cells seeded at a density of 1.0x10^4^in 25μl MEM, 10% FBS in each well of 384-well black plates and incubated for 22-24h. The medium was then removed, and the cell layer rinsed with PBS and incubated with 50µL Glo Lysis buffer (Promega E2661) for 30 min. Then, 25µL of cell lysate was transferred to black low volume 384-well plate, and fluorescence intensity analyzed at 588nm excitation and 635nm emission (SpectraMax Paradigm, Molecular Devices, CA). The 50% and 80% inhibitory concentration (IC_50_ and IC_80_) for each sample were calculated by five-parameter curve fitting and non-linear regression using Labkey Server (Labkey Software). Sera that did not reach 50% or 80% inhibition at the 1:20 dilution were considered to be below the limit of detection and set at half the limit of detection, or 10.

The use of RSV B virus expressing F glycoprotein from the 18537 strain and a G glycoprotein from an A strain for B specific neutralizing assessment is supported by the consistent neutralizing titers of polyclonal sera when comparing this virus and 4 other B reporter viruses (1 laboratory isolate and 3 primary isolates) with matching G glycoproteins from the B strain viruses. The vast majority of neutralizing activity as measured in assays on HEp-2 is directed to the F glycoprotein. The sequence variation in G is in the glycan domains on the N-terminal and C-terminal portions of the molecule. All neutralizing antibodies known to target G recognize a highly conserved central domain surrounding a cysteine noose structure. This structure is highly conserved even between subtypes.

**SUPPLEMENTAL TABLES**

**Table S0. Summary Table of Analyses Performed**

| **RSV Titers** | **Samples Compared** | **Case Population Included** | **Analysis Performed** | **Table Number** |
| --- | --- | --- | --- | --- |
| RSV A/B IC_80_ | Cord blood | All Cases | Unmatched | Table 2 |
| RSV A/B IC_80_ | Cord blood | Strain-specific cases | Unmatched | Table 2 |
| RSV A/B IC_80_ | Cord blood | All cases before 90 days | Unmatched | Table 3 |
| RSV A/B IC_80_ | Month 3 | All cases after 90 days | Unmatched | Table 3 |
| RSV A/B IC_80_ | Cord blood | Cases before 90 days | Matched | Table 4 |
| RSV A/B IC_80_ | Month 3 | Cases after 90 days | Matched | Table 4 |
| Dichotomized RSV A/B IC_80_ | Cord blood | Cases before 90 days | Matched, dichotomized titers | Table 5 |
| Dichotomized RSV A/B IC_80_ | Month 3 | Cases after 90 days | Matched, dichotomized titers | Table 5 |
| RSV A/B IC_50_ | Cord blood | All Cases | Unmatched | Table S3 |
| RSV A/B IC_50_ | Cord blood | Strain-specific cases | Unmatched | Table S3 |
| RSV A/B IC_80_ | Cord blood | Strain-specific cases before 90 days | Unmatched | Table S4 |
| RSV A/B IC_80_ | Month 3 | Strain-specific cases after 90 days | Unmatched | Table S4 |
| RSV A/B IC_50_ | Cord blood | All cases before 90 days | Unmatched | Table S5 |
| RSV A/B IC_50_ | Month 3 | All cases after 90 days | Unmatched | Table S5 |
| RSV A/B IC_80_ | Cord blood | Cases before 90 days | Matched | Table S6 |
| RSV A/B IC_80_ | Month 3 | Cases after 90 days | Matched | Table S6 |
| Dichotomized RSV A/B IC_50_ | Cord blood | Cases before 90 days | Matched, dichotomized titers | Table S7 |
| Dichotomized RSV A/B IC_50_ | Month 3 | Cases after 90 days | Matched, dichotomized titers | Table S7 |
| RSV A/B IC_80_ | Maternal | Strain-specific cases | Unmatched | Table S8 |
| RSV A/B IC_80_ | Maternal | All cases | Unmatched | Table S8 |
| RSV A/B IC_80_ | Maternal | Strain-specific cases | Matched | Table S9 |
| RSV A/B IC_80_ | Maternal | All cases | Matched | Table S9 |
| RSV A/B IC_80_ | Maternal | Strain-specific cases before 90 days | Matched | Table S9 |
| RSV A/B IC_80_ | Maternal | All cases before 90 days | Matched | Table S9 |

**Table S1. Antibody transfer from mother to infant shown in Infant:mother antibody ratio. Rho for correlation between infant and mother antibody level.**

| **Group** | **Median** | **Interquartile Range** | | **Rho** |
| --- | --- | --- | --- | --- |
| **RSV Cases** |  |  |  |  |
| **All Cases A IC_80_(n = 136)** | 1.03 | 0.75 | 1.40 | 0.74 |
| **All Cases B IC_80_(n = 136)** | 1.01 | 0.78 | 1.33 | 0.85 |
| **RSV-A Cases A IC_80_ (n = 22)** | 1.03 | 0.79 | 1.35 | 0.68 |
| **RSV-B Cases B IC_80_ (n = 115)** | 1.04 | 0.78 | 1.37 | 0.86 |
| **ILI Controls** |  |  |  |  |
| **A IC_80_ (n = 180)** | 1.01 | 0.84 | 1.35 | 0.83 |
| **B IC_80_ (n = 182)** | 0.93 | 0.70 | 1.23 | 0.91 |
| **Healthy Controls** |  |  |  |  |
| **A IC_80_ (n = 241)** | 1.15 | 0.85 | 1.42 | 0.84 |
| **B IC_80_ (n = 238)** | 1.07 | 0.80 | 1.40 | 0.85 |

**Table S2. Geometric mean and standard deviation of RSV A and RSV B IC80 by group at zero, three, and six months.**

| **Group** | **Cord Blood** | **3 Months** | **6 Months** |
| --- | --- | --- | --- |
| **RSV-A IC_80_, geo mean (SD)** |  | | |
| **RSV-A Cases** | 226 (2.6) | 75 (2.4) | 116 (2.3) |
| **RSV-B Cases** | 214 (2.3) | 82 (2.2) | 96 (2.3) |
| **ILI Controls** | 247 (2.3) | 59 (1.9) | 55 (2.4) |
| **Healthy Controls** | 264 (2.3) | 62 (3.6) | 57 (4.9) |
| **RSV-B IC_80_, geo mean (SD)** |  | | |
| **RSV-A Cases** | 83 (2.5) | 56 (2.0) | 70 (1.7) |
| **RSV-B Cases** | 83 (2.3) | 59 (2.6) | 91 (2.6) |
| **ILI Controls** | 84 (2.3) | 39 (1.7) | 60 (2.2) |
| **Healthy Controls** | 98 (2.2) | 29 (1.9) | 33 (3.1) |

**Table S3. Cord blood samples, median and IQR of RSV A IC50 and RSV B IC50 by group, with controls compared both to all RSV cases and to strain-specific RSV cases**

| **Group** | **All Cases** | | **Strain Specific Cases** | |
| --- | --- | --- | --- | --- |
|  | **Cord blood IC_50_ median (IQR)** | **p-value (Wilcoxon)** | **Cord blood IC_50_ median (IQR)** | **p-value (Wilcoxon)** |
| **RSV-A IC_50_ Levels** | | | | |
| **RSV Cases** | 886 (435, 1387) | Ref | 803 (319, 1664) | Ref |
| **ILI Controls** | 884 (493, 1626) | 0.48 | 884 (493, 1626) | 0.39 |
| **Healthy Controls** | 939 (564, 1623) | 0.12 | 939 (564, 1623) | 0.20 |
| **RSV-B IC_50_ Levels** | | | | |
| **RSV Cases** | 217 (113, 505) | Ref | 218 (111, 504) | Ref |
| **ILI Controls** | 240 (124, 488) | 0.48 | 240 (124, 488) | 0.49 |
| **Healthy Controls** | 288 (139, 502) | 0.07 | 288 (139, 502) | 0.08 |

*ILI Controls and healthy controls were compared to RSV cases in unmatched preliminary analysis using non-parametric Wilcoxon rank-sum tests.

**Table S4. Infant samples, antibody levels before ILI stratified by timing of ILI (comparing controls to strain specific cases)**

| **Group** | **Cases in first 3 months** | | **Cases after 3 months** | |
| --- | --- | --- | --- | --- |
|  | **Cord blood IC_80_, median (IQR)** | **P (Wilcoxon)** | **3 Month IC_80_, median (IQR)** | **P (Wilcoxon)** |
| **RSV-A IC_80_ Levels** | | | | |
| **RSV-A Cases** | 133.0 (107.9, 170.3) | Ref (n = 10) | 64.8 (44.8, 98.3) | Ref (n = 17) |
| **ILI Controls** | 241.2 (134.0, 485.4) | 0.07 | 52.5 (37.9, 91.4) | 0.38 |
| **Healthy Controls** | 259.0 (143.0, 447.0) | 0.02 | 58.9 (40.3, 99.4) | 0.75 |
| **RSV-B IC_80_ Levels** | | | | |
| **RSV-B Cases** | 82.5 (44.6, 231.0) | Ref (n = 38) | 33.1 (26.1, 47.6) | Ref (n = 5) |
| **ILI Controls** | 75.6 (43.4, 155.9) | 0.45 | 34.8 (27.1, 46.2) | 0.99 |
| **Healthy Controls** | 88.9 (51.3, 163.2) | 0.96 | 39.2 (26.9, 57.5) | 0.25 |

**Table S5. Antibody levels before ILI stratified by timing of ILI (comparing controls to all RSV cases)**

| **Group** | **Cases up to 90 days** | | **Cases after 90 days** | |
| --- | --- | --- | --- | --- |
|  | **Cord blood IC_50_, median (IQR)** | **P (Wilcoxon)** | **3 Month IC_50_, median (IQR)** | **P (Wilcoxon)** |
| **RSV-A IC_50_ Levels** | | | | |
| **All RSV Cases** | 760 (386, 1211) | Ref (n = 47) | 194 (129, 342) | Ref (n = 93) |
| **ILI Controls** | 884 (493, 1626) | 0.24 | 184 (108, 289) | 0.19 |
| **Healthy Controls** | 939 (564, 1623) | 0.08 | 184 (130, 341) | 0.76 |
| **RSV-B IC_50_ Levels** | | | | |
| **All RSV Cases** | 273 (114, 582) | Ref (n = 47) | 63.9 (47, 109) | Ref (n = 93) |
| **ILI Controls** | 240 (124, 488) | 0.67 | 69.9 (47, 130) | 0.73 |
| **Healthy Controls** | 288 (139, 502) | 0.79 | 83.3 (52, 128) | 0.05 |

*ILI Controls and healthy controls were compared to RSV cases in unmatched preliminary analysis using non-parametric Wilcoxon rank-sum tests.

**Table S6. Infant samples IC_80_, matched analysis using conditional logistic regression for odds of being a strain-specific case stratified by timing of case**

| **Group** | **Case Before 3 Months** | | **Case After 3 Months** | |
| --- | --- | --- | --- | --- |
|  | **Estimate** | **P** | **Estimate** | **P** |
| **Comparing RSV A IC_80_** | | | | |
| **RSV A Cases** | 1.00 (n = 10) | Ref | 1.00 (n =17) | Ref |
| **ILI Controls** | 1.22 (0.47, 3.19) | 0.68 | 0.29 (0.06, 1.42) | 0.13 |
| **Healthy Controls** | 1.95 (0.69, 5.45) | 0.20 | 1.05 (0.55, 2.02) | 0.87 |
| **Comparing RSV B IC_80_** | | | | |
| **RSV B Cases** | 1.00 (n = 38) | Ref | 1.00 (n = 54) | Ref |
| **ILI Controls** | 0.70 (0.40, 1.21) | 0.20 | 0.64 (0.26, 1.56) | 0.33 |
| **Healthy Controls** | 1.15 (0.66, 2.01) | 0.62 | 0.95 (0.41, 2.24) | 0.91 |

**Table S7. Infant samples, matched analysis using conditional logistic regression, stratified by timing of case, IC50 dichotomized high/low. High RSV A IC50 = > 855, High RSV B IC50 = > 225.**

| **Group** | **Case Before 3 Months** | | **Case After 3 Months** | |
| --- | --- | --- | --- | --- |
|  | **Odds Ratio* (95% CI)** | **P** | **Odds Ratio* (95% CI)** | **P** |
| **Comparing RSV-A IC_50_** | | | | |
| **All RSV Cases** | 1.00 | Ref (n = 47) | 1.00 | Ref (n = 93) |
| **ILI Controls** | 1.94 (0.76, 4.93) | 0.16 | 0.14 (0.01, 1.27) | 0.08 |
| **Healthy Controls** | 3.25 (1.34, 7.85) | 0.009 | 0.93 (0.26, 3.35) | 0.91 |
| **Comparing RSV-B IC_50_** | | | | |
| **All RSV Cases** | 1.00 | Ref (n = 47) | 1.00 | Ref (n = 93) |
| **ILI Controls** | 0.73 (0.31, 1.72) | 0.47 | 0.39 (0.15, 1.03) | 0.06 |
| **Healthy Controls** | 3.42 (1.27, 9.18) | 0.015 | 0.53 (0.21, 1.32) | 0.17 |

* Odds ratio for having high IC50 among controls compared to all RSV cases

**Table S8. Unadjusted analysis for association between IC_80_ levels from maternal samples at delivery and case/control status.**

| **Group** | **Stratified by A/B case** | | **All Cases** | |
| --- | --- | --- | --- | --- |
|  | **Maternal IC_80_, median (IQR)** | **p-value (Wilcoxon)** | **Maternal IC_80_, median (IQR)** | **p-value (Wilcoxon)** |
| **RSV A IC_80_ Levels** | | | | |
| **RSV Cases** | 209 (104, 516) | Ref (n = 22) | 215 (119, 367) | Ref (n = 142) |
| **ILI Controls** | 234 (150, 385) | 0.49 | 234 (150, 385) | 0.08 |
| **Healthy Controls** | 233 (147, 377) | 0.63 | 233 (147, 377) | 0.18 |
| **RSV B IC_80_ Levels** | | | | |
| **RSV Cases** | 64 (44, 128) | Ref (n = 121) | 64 (44, 128) | Ref (n = 142) |
| **ILI Controls** | 83 (54, 162) | 0.01 | 83 (54, 162) | 0.01 |
| **Healthy Controls** | 82 (50, 156) | 0.05 | 82 (50, 156) | 0.04 |

*ILI Controls and healthy controls were compared to RSV cases in unmatched preliminary analysis using non-parametric Wilcoxon rank-sum tests.

**Table S9. Adjusted analysis using conditional logistic regression for association between IC_80_ levels from maternal samples delivery and case/control status.**

| **Group** | **Stratified by A/B case** | | **All Cases** | |
| --- | --- | --- | --- | --- |
|  | **Odds Ratio** | **p-value** | **Odds Ratio** | **p-value** |
| **Comparing RSV A IC_80_** | | | | |
| **RSV Cases** | 1.00 | Ref (n = 22) | 1.00 | Ref (n = 142) |
| **ILI Controls** | 0.96 (0.44, 2.09) | 0.91 | 1.35 (0.96, 1.90) | 0.08 |
| **Healthy Controls** | 0.81 (0.41, 1.59) | 0.54 | 1.25 (0.92, 1.70) | 0.15 |
| **Comparing RSV B IC_80_** | | | | |
| **RSV Cases** | 1.00 | Ref (n = 121) | 1.00 | Ref (n = 142) |
| **ILI Controls** | 1.17 (0.80, 1.72) | 0.41 | 1.31 (0.80, 1.60) | 0.49 |
| **Healthy Controls** | 1.26 (0.89, 1.79) | 0.20 | 1.24 (0.89, 1.72) | 0.20 |

**Table S10. Maternal samples, matched analysis using conditional logistic regression comparing RSV IC_80_ among controls to cases stratified by RSV strain and all cases before three months of age.**

| **Group** | **Stratified by A/B case** | | **All Cases** | |
| --- | --- | --- | --- | --- |
|  | **Odds Ratio** | **p-value** | **Odds Ratio** | **p-value** |
| **Comparing RSV A IC_80_** | | | | |
| **RSV Cases** | 1.00 | Ref (n = 7) | 1.00 | Ref (n = 44) |
| **ILI Controls** | 1.92 (0.47, 7.94) | 0.36 | 1.40 (0.83, 2.38) | 0.21 |
| **Healthy Controls** | 1.57 (0.43, 5.76) | 0.50 | 1.49 (0.87, 2.58) | 0.15 |
| **Comparing All RSV B IC_80_** | | | | |
| **RSV Cases** | 1.00 | Ref (n = 37) | 1.00 | Ref (n = 44) |
| **ILI Controls** | 0.74 (0.40, 1.39) | 0.35 | 0.97 (0.55, 1.70) | 0.92 |
| **Healthy Controls** | 1.25 (0.63, 2.48) | 0.53 | 1.54 (0.81, 2.92) | 0.18 |

* Odds ratio for a one log increase in IC80 among maternal antibody level at delivery and case/control status among infants before three months of age.
